# An Overview of Methotrexate Indications in Skin Diseases

**DOI:** 10.3390/medicina60071024

**Published:** 2024-06-21

**Authors:** Mădălina Mocanu, Dorina Procopciuc, Dragoș-Florin Gheucă-Solovăstru, Ioana Adriana Popescu, Doinița Temelie Olinici, Adriana Ionela Pătrașcu, Dan Vâță, Laura Gheucă-Solovăstru

**Affiliations:** 1 Department of Dermatology, “Grigore T. Popa” University of Medicine and Pharmacy, 700115 Iasi, Romania; oana.manolache@yahoo.com (I.A.P.); lsolovastru13@yahoo.com (L.G.-S.); 2 Dermatology Clinic, “St. Spiridon” County Emergency Clinical Hospital, 700115 Iasi, Romania; doryna.cruc@gmail.com (D.P.); doinita.p.olinici@umfiasi.ro (D.T.O.); patrascuai@yahoo.com (A.I.P.); 3 Faculty of Medicine, “Grigore T. Popa” University of Medicine and Pharmacy, 700115 Iasi, Romania; mg-eng-31249@students.umfiasi.ro

**Keywords:** methotrexate, psoriasis, skin diseases, off-label indications

## Abstract

Methotrexate is an immunosuppressive drug with remarkable therapeutic results in the treatment of autoimmune and proliferative skin diseases. Although it has been more than half a century since it was first introduced into the therapeutic arsenal of dermatologists, there are currently no standardized therapeutic protocols regarding the prescription of methotrexate in dermatology, with the exception of psoriasis treatment. This review aims to highlight the indications and benefits of methotrexate beyond psoriasis, with a focus on a wide range of inflammatory, vesiculobullous, and proliferative dermatological pathologies.

## 1. Introduction

The therapeutic properties of methotrexate (MTX) are due to its immunosuppressive and antiproliferative capacities, which are well documented in oncology, immunology, and rheumatology [1].

Classical indications for MTX include a wide variety of inflammatory and oncologic skin diseases, and the studies emphasize the established therapeutic values of MTX for the therapy of psoriasis [2]. Currently, MTX is considered the first therapeutic option in patients with psoriasis who are candidates for systemic treatment, in the absence of specific contraindications.

Regarding the benefits of using MTX in other dermatological diseases aside from psoriasis (pityriasis rubra pilaris, scleroderma, systemic lupus erythematosus, atopy, bullous dermatoses, etc.), there are still many controversies [3,4]. The administration of MTX in a varied range of skin–mucosal conditions is mainly based on the hypothesis that, depending on the dosage of MTX, we can drastically change the therapeutic effect from antiproliferative to anti-inflammatory/immunomodulatory, adapted to the pathology we are addressing [5].

For more than 50 years, MTX has proven to be a safe and effective drug that can also be administrated under ambulatory care, not just in the hospital [1,2]. Starting from the scientific and clinical evidence regarding the mechanism of action, the profile of adverse events, and other key defining factors for this substance (dosage, pharmacokinetics, pharmacodynamics, interactions, etc.), we aim to highlight the extensive indications of MTX in dermatology beyond psoriasis.

## 2. Methotrexate—Pharmacological Aspects

The pharmacological properties of MTX indicate it in the therapy of collagen diseases and neoplastic pathologies [6]. Chemically, MTX is synthesized through methylation of its precursor, aminopterin. This precursor was initially used empirically in 1951 for the treatment of psoriasis and rheumatoid arthritis. The first scientific data regarding the therapeutic benefits of MTX in psoriasis were published in 1958, and, since 1972, it has been approved by the FDA for the therapy of this condition [7].

MTX has a triple-action mechanism: anti-inflammatory, anti-proliferative and immunosuppressive. MTX stops the activity of folinic acid by competitively inhibiting the enzyme dihydrofolate reductase. Consequently, the synthesis of deoxythymidylic acid, involved in the generation of DNA, is inhibited. Research has also demonstrated MTX-mediated effects on T cell proliferation, both in vitro and in vivo [5,8]. In one study, Genestier et al. reported that low-dose MTX decreased the antigen-dependent proliferation of T cells taken from patients with rheumatoid arthritis by inducing their apoptosis. In principle, in high doses, the action of MTX as an antifolate is fully known, but the mechanisms of action at low doses are still subjects of extensive research [9,10].

Adenosine is a substance released from human fibroblasts and endothelial cells, capable of inducing anti-inflammatory effects by reducing the adhesion of neutrophils to fibroblasts. MTX promotes the release of adenosine both in vitro and in vivo, which gives it anti-inflammatory properties [2,11]. Adenosine is a potent anti-inflammatory mediator that acts through interactions with a variety of immune cell subtypes, including neutrophils, macrophages, and T cells, modulating a wide range of physiological functions. Numerous studies have confirmed the importance of adenosine in the anti-inflammatory mechanisms of MTX therapy. Additionally, research conducted on animal models of arthritis has demonstrated that the effects of MTX are blocked by caffeine, an adenosine antagonist. Starting from this mechanism, it seems that low doses of MTX stimulate the release of adenosine in inflamed areas in patients with rheumatoid arthritis [12,13]. The association between high caffeine consumption and poor effects of MTX therapy remains a controversial topic. On the one hand, a study led by Nesher et al. supports this concept; on the other hand, Benito-Garcia et al. consider that the effectiveness of MTX is not diminished by the consumption of adenosine antagonists such as caffeine [14].

MTX is a substance with reducible effects on the decrease in cytokine production, a mechanism that supports the notable therapeutic contribution of MTX in the treatment of psoriasis and other autoimmune diseases characterized by increased secretion of pro-inflammatory mediators [15]. MTX was observed to reduce serum levels of TNF-α, IL-1β, and adhesion molecules (E-selectin and VCAM-1) in synovial biopsies of patients with rheumatoid polyarthritis. MTX treatment reduced the the proportion of T cells type CD 4+, TNF-positive cells in the peripheral blood of patients with rheumatoid arthritis and incresead the number of IL-10-positive T cells. This suppression of cytokines was suggested to be due to the inhibition of de novo synthesis of purines and pyrimidines, as the addition of folic acid or thymidine and hypoxanthine reversed the inhibitory effects of MTX on cytokine production. Inhibition of IL-6 secretion by cultured human monocytes may also represent a short-term anti-inflammatory effect of MTX [12,16].

Matrix metalloproteinases (MMP) are a family of Zn^2+^-dependent extracellular enzymes that play important roles in physiological and pathological tissue remodeling. They are synthesized as inactive proenzymes, activated by proteolytic cleavage with activity regulated by tissue inhibitors (TIMPSs) of MMP. MTX therapy decreased collagenase gene expression in synovial tissues from patients with rheumatoid arthritis. MMP-1, -8, and -13 degrade collagen II and III playing the role of collagenases. IL-1 and TNF-α are potent inducers of MMP gene expression. It has been suggested that the inhibitory effect of MTX on MMP expression is mediated by a reduction in IL-1β, rather than by a direct influence on MMP gene expression [17,18]. Interstitial collagenase (collagenase-1, MMP-1), produced mainly by synovial fibroblasts, is a significant member of the MMP family and mediates the degradation of articular cartilage and synovium. Previous studies have shown that MMP-1 is present in the serum of patients with long-standing rheumatoid arthritis. Stromelysin-1 (MMP-3) is known to degrade components of the extracellular matrix, including proteoglycans, gelatin, fibronectin, laminin, and various types of collagens found in increased amounts in the sera of patients with longstanding rheumatoid arthritis. MTX has been shown to reduce MMP-1 and MMP-3 serum levels. These results highlight that MTX should be used for the therapy of patients with inflammatory bone diseases.

In the treatment of autoimmune inflammatory diseases, MTX is frequently administered orally in a single weekly dose. The use of parenteral MTX, especially as a subcutaneous injection, has been shown to have greater clinical efficacy and improved tolerability compared with the oral form. Subcutaneous administration is currently recommended in cases of insufficient clinical response or oral intolerance [19,20].

After oral administration, MTX is absorbed in the proximal jejunum by a proton-coupled folate transporter protein (PCFT/SLC46A1), which transports reduced folate and MTX. The bioavailability of MTX is relatively high, around 64–90%, varying independently of the dose, with the plateau effect occurring at a dose of 15 mg/week, suggesting the supersaturation of the intestinal transporters. The maximum plasma concentration (C max) varies between 0.3 and 1.6 µmol/L and is reached 0.75–2 h after oral administration. Several studies have demonstrated a higher bioavailability of subcutaneous MTX compared to oral MTX, avoiding the plateau effect [21,22].

Approximately 50% of circulating MTX is bound to plasma proteins; in the first hepatic passage, it is metabolized into 7-hydroxymethotrexate (7-OH-MTX), the main metabolite of MTX, and excreted renally. A small amount is excreted through the bile, and some returns to the entero-hepatic circuit. In patients with impaired renal function, ascites, and pleurisy, elimination is reduced, and toxicity monitoring must be carried out by reducing the dose; in some cases, it is even necessary to stop the treatment. The half-life (T 1/2) for small doses is from 4.5 to 10 h [23]. The intracellular transfer of MTX is mediated by a transmembrane reduced folate carrier protein 1 (RFC1/SLC19A1). Inside the cell, some of the MTX is converted to MTX-polyglutamate by an enzyme called folylpolyglutamate synthetase (FPGS), which adds up to seven glutamate groups. It seems clear that, at high doses, the chief effect of MTX is its antifolate effect, but at low doses, MTX has potent anti-inflammatory actions that appear to be mediated via pathways separate from folate antagonism. It has been suggested that the effects of low-dose MTX may be due more to the formation of intracellular polyglutamates and the increased formation of adenosine, a potent endogenous anti-inflammatory mediator. The serum half-life is 6–7 h for MTX, but much longer for polyglutamate derivatives, resulting in the possibility of weekly administration, despite the short half-life. MTX-polyglutamate has been highlighted in erythrocytes, neutrophils, and monocytes, and is considered to be the active form of MTX [2,24].

Adverse reactions have been reported in approximately 30% of patients treated with MTX. The toxic effect is generally moderate and the mortality rate is low (1.2 out of 100,000 cases). The toxicity of MTX depends on the dose, the duration of the treatment, and the co-morbidities of the patient. The administration of MTX is often associated with multiple adverse events, among which gastrointestinal toxicity is very frequent. MTX can induce malabsorption, diarrhea, severe pain, and, in some cases, mucositis, which can affect the intestinal area or oral mucosa. Mucosal alteration promotes the recruitment of mononuclear phagocytes with proinflammatory effects, due to varied released cytokines. This fact additionally stimulates tissue alteration. Although several studies have suggested the disruption of immune regulation initiated by direct MTX-induced injury, the exact pathogenic mechanism underlying the MTX-induced intestinal/oral mucositis still remains unknown [25,26].

Severe adverse effects are usually associated with predisposing factors (hepatic, renal, or hematologic diseases), dosage errors, and lack of patient compliance. Table 1 lists the main adverse reactions induced by MTX [1].

Data in the literature regarding the need for folic acid supplementation in patients treated with MTX are lacking and varied. Some studies have demonstrated the beneficial effects of folic acid in combating gastrointestinal adverse effects of MTX, such as nausea or loss of appetite. Another analysis reviewing seven randomized controlled trials found that supplementation with folic or folinic acid reduced the incidence of MTX-related gastrointestinal or mucocutaneous adverse events by 79% or 42%. Folic acid can decrease the effectiveness of MTX in a context in which the combination of controlled doses is recommended. These range predominantly between 1 and 5 mg folic acid, with most dermatologists opting for supplementation with folic acid 5 mg/day, except on the day that MTX is administered [27,28,29,30].

There are a number of relative or absolute contraindications regarding the administration of MTX that we present in Table 2 [31].

## 3. Methotrexate—Dermatological Indications

The complex mechanism of action of MTX, depending on the dose and rate of administration, gives it therapeutic value in numerous pathologies, but we will focus on the indications of this drug in dermatological conditions. Numerous studies have demonstrated its effectiveness in other dermatological conditions, such as atopic dermatitis, chronic urticaria, mycosis fungoides, bullous dermatoses, connective tissue diseases (systemic lupus erythematosus, systemic sclerosis, morphea, and dermatomyositis), keratoacanthoma, and pityriasis rubra pilaris [5]. The most common indications of MTX in dermatology are presented in Table 3 [32,33]. They were divided into two categories that refer to the evidence of scientific data regarding the effectiveness of MTX in the therapy of certain cutaneous diseases.

### 3.1. Psoriasis

MTX is an appropriate choice for patients with moderate–severe forms of psoriasis, unresponsive or with incomplete response to topical therapies, oral retinoids (Acitretin), cyclosporine, narrow-band UVB, and psoralen/UVA phototherapy. It is also indicated for patients who do not tolerate the therapies mentioned above, or can be associated with biological therapies when the response to the biological treatment does not fall within the target therapy [20,21].

The evaluation prior to initiation of MTX therapy includes the patient’s medical history, local and general clinical examination, histopathological confirmation of the disease, and paraclinical examinations. Basic laboratory investigations refer to the complete blood count, analysis of liver and kidney functions, serology for hepatitis B and C, screening to exclude latent or active tuberculosis, and pregnancy testing in fertile women. In addition, patients are advised to use contraceptive methods during MTX treatment and for 3–6 months after its completion. According to the British Society for Rheumatology guidelines on prescribing drugs during pregnancy and breastfeeding, doses of MTX lower than 25 mg/week administered to men do not present a teratogenic risk [34].

The consumption of alcohol or other hepatotoxic substances is totally prohibited during the administration of MTX [5,9].

MTX is incorporated in two pharmaceutical forms: tablets or injectable solution in a prefilled syringe. The recommended starting dose is between 10 and 20 mg/week, if the patient has no risk factors for adverse effects (impaired renal function, risk of hepatotoxicity, or drug interactions). The treatment will be started with a lower dose (5–7.5 mg/week) and the patient will be evaluated monthly (clinically and biologically) if any of these risk factors are present. For patients without comorbidities, monthly evaluation is recommended during the first two months of treatment, then every 3 months.

We emphasize that the minimum effective dose of MTX in most patients with psoriasis is 7.5 mg/week, except in cases where creatinine clearance is reduced [22,23]. Also, the average dose that provides long-term therapeutic effects in the absence of adverse reactions is 15 mg MTX/week. The manner of tapering the dose of MTX in patients with psoriasis who have achieved a satisfactory therapeutic response is still debated in the literature, but most guidelines call for de-escalation of the dose by 2.5 to 5 mg every 2 to 4 months [1,24].

### 3.2. Atopic Dermatitis

Atopic dermatitis is a chronic inflammatory dermatosis with variable degrees of extension and severity that can affect newborns, children, or adults [35]. Although the majority of patients respond favorably to treatment with topical corticosteroids and immunomodulators associated with oral antihistamines, approximately 10% remain refractory to these conventional treatment modalities. In such cases, a number of options, like phototherapy or systemic immunomodulatory agents (corticosteroids, cyclosporine, azathioprine, mycophenolate, or biological agents such as dupilumab) could be considered.

Recently, MTX has become a reliable option for the treatment of adult atopic dermatitis. It is used as an off-label systemic alternative, with limited randomized controlled or cohort studies on the effectiveness of MTX in atopy. Existing research results have shown varied clinical responses, with percentages ranging from 53.85% to 93% of patients with more than 75% clinical improvement achieved within 4–12 weeks of MTX treatment. Adverse effects were reported in 34.62% of patients, predominantly gastrointestinal (nausea, vomiting, and increased serum transaminases), and hematological events, such as bone marrow inhibition, were reported extremely rarely [35,36,37].

Other studies have revealed the remarkable impact of MTX therapy in atopy in terms of significant improvement in quality of life, with a greater than 50% decrease in DLQI for half of the patients included in the study. In addition, scientific evidence emphasizes the effectiveness of MTX in low doses (7.5 mg/week) in the long term, or for short periods during the period of disease activity (usual dose 15 mg/week). Also, intramuscular MTX appears to induce faster responses and a more prominent decrease in SCORAD and DLQI than oral treatment [35,36].

Cyclosporine is a systemic immunosuppressant drug recommended in severe forms of atopic dermatitis. Studies have shown that increasing the dose of MTX to 25 mg/week for 20 weeks implies a superior efficiency and a greater safety profile compared to cyclosporine. However, additional research is needed to certify MTX as a first-line therapy for severe, refractory forms of atopic dermatitis [37,38].

Regarding the pediatric population, the benefit–risk balance for the use of MTX in children and adolescents with atopy remains debatable. Anderson et al. performed a retrospective study on 55 children with atopic dermatitis, treated with low-dose MTX (0.45 mg/kg weekly) for 15.3 months, showing a clinical improvement of 76.4%. The most common adverse effects were gastrointestinal, acquired skin infections, and hematological abnormalities [39,40,41].

### 3.3. Chronic Urticaria

Chronic urticaria is a frequent pruritic dermatosis characterized by the persistence of a papular rash for at least 6 weeks. The typical skin lesions of urticaria are erythematous papules of different sizes, with a well-demarcated polycyclic outline, pink–red, with a tendency to discoloration in the center; they are smooth, soft to the touch, edematous, transient, isolated or confluent in plaques, and very itchy.

First-line treatment for chronic urticaria includes oral antihistamines, but, despite their proven efficacy, approximately 36.8% of patients do not have an adequate response to treatment and require additional therapy. MTX it is a therapeutic alternative option for chronic recalcitrant urticaria, due to its anti-inflammatory properties. The effect of MTX on adenosine and its capacity to inhibit cytokines, leucocyte chemotaxis, and free radicals leads to the improvement of skin lesions from chronic urticaria. A recent meta-analysis, based on data published in the literature between 2000 and 2021, which studied the effectiveness of immunosuppressive therapy in patients with refractory chronic urticaria, highlighted the fact that the therapeutic action of MTX proved to be weaker in patients with chronic urticaria compared to placebo [42,43]. The current recommendation in urticaria does not detail the dose, nor duration treatment, with MTX. From this point of view, it does not yet exist in large studies dedicated to MTX treatment for chronic urticaria [44].

Several case reports and case series that included patients with chronic urticaria unresponsive to classical treatment showed that low doses of MTX (10–15 mg/week) led to complete disease resolution in 87% of subjects. Perez et al. evaluated 26 patients with chronic corticosteroid-dependent urticaria treated with MTX; 12 of 16 patients had a complete response to MTX, and, in 7 patients, the doses of oral corticosteroids could be reduced. Another recent study showed that MTX appears to be a useful option in the therapy of chronic urticaria, both as a monotherapy and for combination therapy in patients resistant to Omalizumab and high-dose antihistamines [44,45]. MTX certainly improves the quality of life in patients with refractory chronic urticaria, primarily by reducing pruritic symptoms, but further evidence is needed to introduce it into standardized urticaria treatment guidelines.

### 3.4. Systemic Lupus Erythematosus

A double-blind, placebo-controlled study that included 41 patients showed that MTX can be used successfully in patients with systemic lupus erythematosus (SLE), allowing the disease to be controlled and the dose of oral corticosteroids to be reduced. Also, the same study showed a significant reduction in disease activity index in cases treated with MTX (20 patients) compared to the placebo group (17% vs. 84%) after 6 months of treatment with MTX 15–20 mg/week. Two single-center retrospective studies reported the effectiveness of MTX in the treatment of discoid cutaneous and refractory systemic lupus. Lehman et al. suggested that the use of MTX in pediatric SLE should be reserved for patients with severe nephritis who are resistant to cyclophosphamide [46,47,48].

European guidelines list MTX as a second-line, and Mycophenolate as a third-line, therapy indicated for patients with cutaneous lupus erythematosus refractory to antimalarial drugs. The results of the related studies suggest comparable response rates between MTX and Mycophenolate, but further research is necessary [49].

SLE-related mortality is closely related to lupus nephritis and neuropsychiatric impairment. The way in which MTX is effective in the neurological therapy of SLE refers to the fact that intrathecal MTX reduces the pressure of the cerebrospinal fluid and lowers the levels of spinal proteins through its anti-inflammatory effects. The possibility of intrathecal administration of MTX prevents the occurrence of vital complications in patients with SLE and increased levels of proteins in the cerebrospinal fluid [50].

### 3.5. Bullous Dermatoses

Autoimmune bullous diseases represent a heterogeneous group of dermatoses with varied etiopathogenesis, characterized by the appearance of blisters on the skin and mucosa, as a result of a loss of cell adhesion. The most common bullous diseases encountered in dermatological practice are pemphigus vulgaris and bullous pemphigoid. Topical therapy with super potent corticosteroids is the first line of treatment for mild and moderate bullous pemphigoid, followed by oral corticosteroids (at an initial dose of 0.5 mg/kg/day Prednisone or Prednisolone). Second-line therapy refers to the administration of Doxycycline or Dapsone. Immunosuppressive medication (azathioprine, cyclosporine, mycophenolate, or MTX) is indicated in corticosteroid-dependent or relapsing bullous pemphigoid. MTX can be used as an alternative therapy for patients with significant long-term adverse effects or comorbidities that contraindicate oral corticosteroids or other immunosuppressive agents. Patients with recalcitrant bullous pemphigoid can be treated with MTX in combination with other conventional immunosuppressants [51,52].

A recent retrospective review of the English medical literature concluded that MTX is an effective and well-tolerated immunomodulatory agent that allows for the tapering of corticosteroids in patients with bullous pemphigoid and pemphigus vulgaris. MTX may be a viable maintenance therapy once disease remission has been achieved with corticosteroids. Oral lesions often respond more slowly to MTX, while skin effects appear more rapidly. An analysis of data on 62 patients with bullous pemphigoid found clinical improvement in 59 patients (95%) [53]. McCluskey and colleagues reported that 15 of 17 patients with ocular cicatricial pemphigoid responded to MTX (5–25 mg weekly) alone or in combination with corticosteroids [54].

### 3.6. Localized and Systemic Sclerosis

Localized scleroderma (morphea) is a connective tissue disease of unknown etiology, characterized by skin fibrosis due to excessive collagen synthesis in a certain area. The condition has various clinical forms: in plaques, linear, frontoparietal lesions “en coup de sabre”, or generalized morphea. MTX (15–20 mg/week) is used in the treatment of all forms of localized scleroderma, usually in combination with systemic corticosteroids [55]. A recent consensus guideline study established by Trials Consortium (SCTC) and the Canadian Scleroderma Research Group (CSRG) found that 62% of scleroderma experts use MTX as a first-line treatment of diffuse skin thickening, and 60% use MTX as a first-line treatment for inflammatory arthritis [32].

MTX is established as the most frequently used immunosuppressive agent in the treatment of localized scleroderma in children. Low-dose MTX can be combined with systemic corticosteroids for rapidly progressive and disabling localized scleroderma in children. MTX ameliorates skin fibrosis by direct action on epithelial fibroblasts and by its anti-inflammatory effects. Uziel et al. demonstrated the efficacy of combined treatment with MTX (0.3–0.6 mg/kg week) and Methylprednisolone IV (30 mg/kg/day) on 3 consecutive days/month for 3 months in 10 children with localized scleroderma. Christen-Zaech et al. analyzed 136 children with localized scleroderma, of whom 39 received MTX. Most of these patients responded well to MTX therapy, with a marked reduction in skin induration. Another randomized, double-blind, placebo-controlled trial of MTX (15 mg/m^2^/week for 12 months) demonstrated the efficacy and safety of MTX in the treatment of juvenile localized scleroderma [56,57].

Systemic sclerosis is a chronic autoimmune condition that affects the skin and viscera, leading, over time, to generalized fibrosis. MTX is considered the most efficient treatment for reducing skin fibrosis, although the effects on other affected areas have not been demonstrated.

### 3.7. Cutaneous Sarcoidosis

Methotrexate is the drug of choice in the second-line treatment of sarcoidosis in both adults and children. It is reserved for the treatment of refractory sarcoidosis to systemic steroids in patients who have developed steroid-related adverse reactions, as well as in patients with severe organ failure. MTX (10–15 mg/week) can be combined with systemic steroids or used as a monotherapy. Various authors have reported improvements in skin lesions with MTX. However, relapse can occur when MTX is stopped, suggesting that MTX controls, but does not cure, the disease [58,59].

### 3.8. Mycosis Fungoides

Mycosis fungoides is a cutaneous T cell lymphoma that has been treated with MTX for many years, although data on the therapeutic benefits of MTX in this setting are limited. The mechanism of action of MTX in mycosis fungoides is still unclear, but it seems that the cytostatic effects are mainly involved and, secondarily, the anti-inflammatory and immunomodulatory ones [60].

MTX is usually administered orally as a second-line therapy, according to the European Organization for Research and Treatment of Cancer and the World Health Organization recommendations for stages IA, IB, and IIA of mycosis fungoides [61]. The dose used is 20–75 mg/week, divided into 3 doses every 12 h, and can be associated with systemic corticosteroids, PUVA phototherapy, or Interferon.

Zackheim et al. [62] studied 69 patients with mycosis fungoides, initially treated with MTX (average dose 25 mg/week) without significant clinical response. Increasing the dose of MTX to a maximum of 75 mg/week improved the appearance of the lesions, thus reaching the conclusion that the efficacy of MTX is dose-dependent [50]. Also, Olek-Hrab et al. analyzed the therapeutic response of MTX in 79 patients treated with doses of MTX between 25 and 75 mg/week, reporting that remission was achieved in 70% of patients within 1–3 months. Longer periods of remission were associated with longer durations of treatment, which implied a higher rate of side effects. The tapering of MTX doses in patients with mycosis fungoides is guided by clinical response and hematological monitoring. Combination chemotherapy regimens, including higher-dose intravenous MTX with Leucovorin, have been used in advanced disease [62,63].

### 3.9. Pityriasis Rubra Pilaris

Pityriasis rubra pilaris is a hereditary or acquired chronic inflammatory dermatosis, characterized by isolated or plaque-forming keratotic papules that sometimes progress to erythroderma. The use of MTX for pityriasis rubra pilaris is strongly supported by its mechanism of action as an antiproliferative and anti-inflammatory agent. A literature review including 116 cases reported complete remission in 23.3% of patients and significant clinical improvement in 17.2% of subjects. Clayton reported the success of combination therapy of MTX with oral retinoids in refractory pityriasis rubra pilaris. However, this combination should be used with caution, as it increases the risk of liver toxicity [64]. In juvenile pityriasis rubra pilaris, Klein et al. recommend the use of MTX only in severe recalcitrant cases [65,66].

### 3.10. Keratoacanthoma

Keratoacanthoma is a skin tumor with low malignancy potential that affects especially the elderly and areas exposed to the sun. Histologically, it is a subtype of well-differentiated squamous carcinoma, or a squamous lesion with spontaneous involution, originating in the pilosebaceous follicles. The classic clinical form is that of a well-defined erythematous nodule with a typical keratosis crateriform central area. Other particular manifestations refer to giant, eruptive keratoacanthomas and centrifugum marginatum lesions. The curative treatment is surgical excision, but, in the cases of large lesions, it is preferable to reduce them by administering intralesional MTX prior to the surgical procedure.

MTX is preferred for facial lesions, with the goal of reducing post-excisional skin defects [67]. MTX is indicated “off-label” in the local therapy of keratoacanthoma, based on its properties to inhibit the growth of benign skin tumors. The dosage provides MTX intratumoral injection in four quadrants, in 1 mL of solution of different concentrations (5, 12.5, and 25 mg/mL), at intervals of 14–21 days. Positive effects, such as reducing the size of the keratoacanthoma, are established after 1–4 injections. In the literature, there are only a few case series on this subject, with 38 treated patients, of whom 92% reportedly experienced a complete therapeutic response [5].

### 3.11. Other Indications of MTX in Dermatology

In recent years, a number of studies have been conducted that provide increasingly consistent scientific evidence regarding the benefits of extensive use of MTX in dermatology. Some of the dermatoses responsive to off-label MTX therapy are alopecia areata, universal or total, vulvovaginal erosive lichen planus, pityriasis lichenoides, reticulohistiocytosis, vitiligo, Behcet’s disease, and Reiter’s syndrome [68,69].

In an era of continuous expansion of many innovative molecules intended for the therapy of dermatological or systemic diseases, studies have demonstrated, in addition to optimal efficacy and safety parameters, significantly lower costs for MTX. Therefore, in addition to its wide range of indications, MTX represents an optimal treatment alternative from a pharmacoeconomic point of view [70].

## 4. Conclusions

Methotrexate is a drug that has been traditionally used in dermatology for more than half a century. Although the indication of choice for MTX is psoriasis, it is currently used with success in other skin–mucosal dermatological pathologies, where it stands out thanks to the therapeutic benefits it offers. The main dermatoses in which MTX is recommended, with the exception of psoriasis, are atopic dermatitis, chronic urticaria, mycosis fungoid, pityriasis rubra pilaris, vesiculobullous diseases, skin carcinomas, lupus erythematosus, and scleroderma. The mechanisms of action of MTX are well known, and their complexity offers new therapeutic perspectives in cutaneous and extra-cutaneous pathologies.

## Figures and Tables

**Table 1:** Adverse effects of methotrexate.

Tissue/Organ	Side Effect
Systemic	Fatigue
	Malaise
	Anorexia
Gastrointestinal	Diarrhea
	Aphthous stomatitis
	Hepatotoxicity
	Dyspepsia
	Abdominal pain
	Indigestion
	Nausea
Hematological	Bone marrow aplasia (anemia, leukopenia, and thrombocytopenia)
Reproductive	Teratogenicity
	Oligospermia
Pulmonary	Pneumonitis
	Pulmonary fibrosis
Mucocutaneous	Mucositis
	Photosensitivity
	Drug hypersensitivity reaction
	Diffuse non-inflammatory alopecia
	Squamous cell carcinoma
Infections	Opportunistic infections
	Tuberculosis reactivation
	Hepatitis
Neurological	Headache
	Fatigue
	Mood alterations

**Table 2:** Contraindications to the use of methotrexate.

Absolute Contraindications	Relative Contraindications
Pregnancy/breastfeeding in women/conception in men	Renal insufficiency (reduce dose if creatinine clearance is between 20 and 50 mL/min)
Marked anemia, leukopenia, or thrombocytopenia	Persistently abnormal liver enzymes
Alcohol abuse	Chronic viral hepatitis without evidence of significant liver disease
Acute peptic ulcer	Family history of inheritable liver disease
Severe respiratory failure	Obesity (body mass index greater than 30)
Immunodeficiency	Diabetes mellitus
Active infectious disease, especially active untreated tuberculosis or HIV infection	History of significant exposure to hepatotoxic drugs (e.g., azathioprine, retinoids, sulfasalazine) or chemicals
Liver disease (active or recurrent hepatitis, hepatic fibrosis, or cirrhosis on liver biopsy)	Steatohepatitis
Recent vaccination, especially with live vaccine	Untreated hyperlipidemia
Hypersensitivity—history of allergic events correlated with MTX administration	Lack of folate supplementation (often due to the patient’s lack of compliance)
Renal insufficiency (creatinine clearance ↓ 20 mL/min)	Age ↓ 3 years

**Table 3:** Common dermatological indications for MTX.

Highest Evidence	Lowest Evidence
Psoriasis vulgaris	Pityriasis rubra pilaris
Skin atopy	Pityriasis lichenoides
Systemic sclerosis	Chronic urticaria
Systemic lupus erythematosus	Bullous dermatoses (pemphigus, pemphigoid, and ocular cicatricial pemphigoid)
Mycosis fungoides	Dermatomyositis
	Polyarteritis nodosa
	Cutaneous sarcoidosis
	Granuloma annulare
	Lymphomatoid papulosis
